# Immunotoxins: From Design to Clinical Application

**DOI:** 10.3390/biom11111696

**Published:** 2021-11-15

**Authors:** Robert J. Kreitman, Ira Pastan

**Affiliations:** Laboratory of Molecular Biology, Center for Cancer Research, National Cancer Institute, National Institutes of Health, Bethesda, MD 20892, USA; pastani@mail.nih.gov

The Special Issue of Biomolecules entitled “Immunotoxins, From Design to Clinical Application” contains seven reviews related to immunotoxins. Molecules that were originally called immunotoxins contained an antibody and a protein toxin chemically conjugated together to target antigen-bearing cells for catalytic destruction [1,2,3,4,5]. Early immunotoxins had non-specific toxicity due to retention of the binding domain of the toxin, until the toxin binding domains were deleted [6,7,8,9]. Advances in recombinant protein engineering and production techniques led to the construction of recombinant growth factor–toxin fusion proteins [10,11,12,13], which are often considered cousins of immunotoxins since they lack an antibody.

Many of these early and more recent molecules are discussed in the review by Antignani et al. from the Lab of Dr. FitzGerald [14], including chimeric toxins targeted by IL2, IL3, IL4, IL6, IL7, and IL13. In 1989, the first recombinant immunotoxin was described, containing the variable domains of the anti-Tac (anti-CD25) Mab fused to truncated pseudomonas exotoxin [15]. This led to LMB-2, which kills CD25+ target cells [16,17] and achieved remissions in patients [18,19]. This, as well as the other reviews, contain excellent introductions for the mechanism of intoxication. Toxins with binding domains deleted originally included deglycosylated ricin A chain (dgA), PE40, or PE38 from pseudomonas exotoxin (PE), and DT388, or DAB389 from diphtheria toxin (DT). As DT and PE are made as single chain proteins in bacteria with proteolytic processing sites to separate the catalytic fragment of the toxin from the binding domain, they are ideal for making recombinant toxins [20]. Recombinant immunotoxins reviewed by Antignani et al. include those targeting epidermal growth factor receptor (EGFR), the tumor specific EGFRvIII, and transferrin receptor. Ligand-toxin chimeric fusions and recombinant immunotoxins were also discussed for Her2, Her3, and the urokinase amino terminal fragment (ATF), C-C Chemokine Receptor Type 9 (CCR9), EPH receptor, and MSH receptors [14]. CCR9, the receptor for chemokine CCL25, is overexpressed on solid tumors of breast, ovary, lung, and prostate, and liver, and on T-cell leukemias, and is associated with metastasis and resistance to chemotherapy [21]. However, the activity of the recombinant toxin CCL25-PE38 was limited by lack of CCR9 in the advancing edge of tumors [22]. Regarding EPH receptors, the review includes a recent report describing an EPHA2-specific scFv connected to PE38KDEL which kills breast cancer cells [23].

The review I contributed with Dr. Pastan focuses on the clinical development of recombinant immunotoxins for hairy cell leukemia (HCL) [24]. HCL expresses high levels of CD25 and CD22, the 2 antigens targeted, and is relatively indolent, allowing treatment cycles to be spaced 4 weeks apart. Several recombinant immunotoxins targeting HCL are reviewed, but the most successful is moxetumomab pasudotox (Moxe), approved by the FDA in 2018 for relapsed/refractory HCL. In pivotal phase 3 testing, it achieved a complete remission (CR) rate of 41%, most without minimal residual disease (MRD), which can lead to relapse. Moxe is the only non-chemotherapy single-agent that eliminates MRD in a significant percentage of HCL patients. Rituximab is currently being tested in combination with Moxe for two goals, firstly to help deplete HCL cells and allow Moxe to bind to the remaining HCL cells, and secondly, to deplete normal B-cells, which produce anti-drug antibodies. As recently reported at the 2021 American Society of Clinical Oncology Annual Meeting, of the first 9 patients receiving Moxe-Rituximab, 7 (78%) achieved MRD-free CR, without dose-limiting toxicity. These results support the evaluation of Moxe-Rituximab for 1st and 2nd line treatment of HCL. We also suggest that Moxe-Rituximab could be useful in the much more common non-Hodgkin’s lymphomas (NHL) with high CD22 expression, such as mantle cell lymphoma, follicular lymphoma, marginal zone lymphoma, lymphoplasmacytic lymphoma, and some cases of chronic lymphocytic leukemia. In these patients who have minimal residual lymphoma cells after definitive immunochemotherapy and would otherwise require chronic maintenance therapy, Moxe might eliminate MRD and result in long-term treatment-free CRs.

To target CD19+ and/or CD22+ lymphomas and leukemias, Dr. Vallera’s group previously engineered bispecific recombinant immunotoxins, notably DT2219, containing both anti-CD22 and anti-CD19 Fv fragments, achieving 1 complete remission (CR) and 2 partial responses (PR) in 18 patients [25,26]. In this issue, Oh et al. in Dr. Vallera’s group contributed an update on the bispecific targeting of EGFR and urokinase receptor (uPAR) using ligand-targeted toxins in solid tumors. The bispecific chimeric toxin contained human EGF and ATF binding to EGFR and urokinase plasminogen activator receptor (uPAR), respectively, and the truncated PE fragment PE38 which ends in Lys-Asp-Glu-Leu (KDEL) [26,27]. The native C-terminus of PE ends in Arg-Glu-Asp-Leu-Lys (REDLK) with the terminal lysine quickly removed by carboxypeptidases in vivo or in tissue culture to result in REDL [28]. Binding studies showed that KDEL binds to the KDEL receptor with ~100-fold higher affinity than REDL, translating to up to ~100-fold improved cytotoxicity when comparing recombinant toxins ending in KDEL vs. REDLK [29]. The bispecific EFGR-uPAR-targeted toxin, abbreviated eBAT, showed antitumor activity in an intracranial U87 orthotopic therapy model [30]. eBAT also achieved significant antitumor activity in human head and neck squamous cell carcinoma [31], triple negative breast cancer, human sarcomas [27], and cancer stem cells. In canine clinical trials, eBAT showed antitumor activity against hemangiosarcoma, and other sarcomas [27]. Prolonged survival in dogs was achieved in combination with chemotherapy [32]. Thus, eBAT is an exciting recombinant toxin due to its bispecific targeting and extremely high cytotoxicity.

Doctors Fleming and Ho describe an excellent update [33] on the preclinical development of recombinant immunotoxins targeted to glypican-3 (GPC3), a 580 amino acid 70 kDa protein that is upregulated in most cases of hepatocellular carcinoma [34,35]. The most promising GPC3 immunotoxins developed by Dr. Ho’s lab were those containing the engineered single-domain antibody HN3 that inhibits Wnt/β-catenin signaling by binding to the Wnt functional binding site on the N-lobe of GPC3 [33,35,36,37,38]. In addition to their unique signaling inhibitory effect, the immunotoxins based on the single-domain antibody have high expression levels and high production yield in *E. coli* [36]. HN3-mPE24 contained a B-cell ‘deimmunized’ toxin missing multiple B-cell epitopes, and a furin-cleavable linker [39]. In HN3-T20, 6 T-cell epitopes in the adenosine diphosphate (ADP) ribosylating domain III of PE were deleted [40]. Despite the mutations, the ADP-ribosylation activity of HN3-mPE24 and HN3-T20 was reduced only 11% and 27%, respectively. HN3-mPE24 had the best in vitro cytotoxicity, while HN3-T20 showed greatest prolongation in overall survival of mice bearing Hep3B xenografts. To increase the half-life, albumin-binding domains from *Streptococcal* protein G (ABD) and llama V_H_H antibody (ALB1) were added to HN3-T20, resulting in a 22-44-fold increase in half-life. HN3-ALB1-T20 is an exciting candidate for the treatment of hepatocellular carcinoma (HCC) due to its potent activity and potential to avoid immunogenicity.

To target solid tumors including ovarian cancer, Mab K1 was isolated [41,42] and found to bind to the antigen mesothelin, a 40 kDa glycoprotein on mesothelial cells, including ovarian cancers, mesotheliomas and pancreatic carcinomas [43,44]. Biodistribution studies in mice bearing human mesothelin+ tumors (A431-K5) showed tumor localization and antibody retention [45]. A chemical conjugate between K1 and LysPE38QQR was highly cytotoxic to A431-K5 cells and achieved CRs in tumor bearing mice [46]. To construct a recombinant immunotoxin, a high-affinity Fv was isolated from DNA-immunized mice after screening by phage display, and the resulting single-chain recombinant immunotoxin SS(scFv)-PE38 was highly active against A431-K5 tumors [47]. SS1P, which has an increase in affinity and a disulfide-stabilized Fv fused to PE38, was tested in two phase 1 trials, one by continuous infusion and one by bolus dosing, with antitumor activity limited by immunogenicity [48,49]. Major regressions in mesothelioma were observed once immunogenicity was blocked with pentostatin and cyclophosphamide, to deplete T and B cells [50]. SS1P was also active when combined with pemetrexed and cisplatin [51]. In the recent review by the group led by Dr. Alewine, a variety of SS1P-based recombinant immunotoxins are reviewed, including LMB-12, LMB-100, LMB-165, and LMB-244 [52]. These molecules contain PE domains 2 and 3 deleted or mutated to decrease immunogenicity, and either antibody constant domains (Fab), albumin binding domain (ABD), or PEGylation added to increase plasma lifetime. LMB-100, containing an SS1(Fab) and a modified version of PE24, has been tested in seven clinical trials, three of them still recruiting patients. For mesothelioma, LMB-100 was initially tested as a single-agent and then combined with nab-paclitaxel based on data showing in vitro synergy. In a subsequent mesothelioma trial, patients received anti-PD-1 Mab pembrolizumab following LMB-100. Patients with PD-L1+ tumors responded, including a CR that remained disease-free after 33 months [53]. In a trial of 17 evaluable patients with pancreatic cancer treated with LMB-100 and nab-paclitaxel, seven (41%) had a >50% decrease in tumor marker CA-19-9, and one had a PR [54]. To enhance efficacy and decrease immunogenicity, a variety of agents to combine to LMB-100 or non-PE containing agents have been suggested and are being studied.

Doctors Dieffenbach and Pastan present a review of mechanisms of resistance to PE, along with ongoing work to overcome resistance [55]. Resistance at the earliest step of intoxication affects binding and internalization and may include antigen downregulation or shedding. The transmembrane glycoprotein TACE was implicated in mesothelin shedding, and knock-down with siRNA or inhibition of TACE increased the sensitivity of cells to SS1P [56]. One potential mechanism of synergy between SS1P and taxol is inhibition of mesothelin shedding by taxol [57]. This led to clinical trials of LMB-100 with nab-paclitaxel. Resistance to the second step of immunotoxin intoxication, proteolytic processing and trafficking, may involve impaired cleavage by Furin. To determine if tyrosine kinases might decrease immunotoxin activity in this manner, siRNA knockdowns were studied for the 87 known tyrosine kinases and several knockdowns were found to be associated with increased immunotoxin efficacy. To prevent destruction of the immunotoxin in lysosomes, another resistance mechanism, choosing an antigen that traffics more quickly to the trans-reticular Golgi rather than lysosomes, and using a lysosomal disrupting agent are possible avenues being studied. Knockdowns of genes encoding endoplasmic reticulum and Golgi proteins were associated with increased sensitivity to SS1P [58]. Failure to bind to the KDEL receptor, which is critical for cytotoxicity [29,59], is also a form of resistance. Resistance of inhibition of protein synthesis may involve the diphthamide residue of elongation factor-2, necessary for ADP ribosylation [60,61] or the collagen-activated tyrosine kinase DDR1. Failure to induce apoptosis might feature anti-apoptotic protein Mcl-1 and degradation of pro-apoptotic BCL-2 proteins Bim and Noxa. The cellular apoptosis susceptibility gene (CAS/CSEL1) is also important in apoptosis from recombinant immunotoxin. Targeting multiple resistance pathways may produce synergistic improvements in immunotoxin targeting, and this important work is continuing.

Dr. Bera contributed an in-depth review on B-cell maturation antigen (BCMA) immunotoxins [62]. BCMA is expressed on multiple myeloma (MM) cells, but not stem cells or memory B-cells. An antibody drug conjugate (ADC) containing an anti-BCMA Mab and monomethyl auristatin F (MMAF) is FDA-approved with the name belantamab mafodotin to treat relapsed/refractory MM [63]. BCMA has been targeted with CAR-T cells, has been given breakthrough therapy designation by the FDA. To target MM with an immunotoxin, the anti-BCMA Mab BM306 was isolated [64]. LMB-70 was then constructed containing BM306(VH-C_H_1) fused to PE24, and this fusion was connected via a disulfide-bond to the BM306 Fd fragment (VL-Ck). A variety of improvements were made to LMB-70, and a total of seven other recombinant immunotoxins were reviewed. The most recent of these is LMB-267, containing the T20 toxin with an A494R mutation [62]. In vivo studies are planned for several of the promising candidates.

In summary, this special issue on immunotoxins contains a general review of immunotoxins and growth-factor fusion toxins [14], a clinically oriented review for recombinant immunotoxin Moxe approved for HCL [24], reviews focusing on targeting both EFGR and uPAR [26], mesothelin-expressing tumors [52], GPC3 targeting in HCC [33], and BCMA targeting in MM [62], and finally resistance mechanisms of immunotoxins [55]. Although the number of targeted therapies is increasing for many types of tumors, recombinant immunotoxins have a special advantage due to their extreme potency, and it is anticipated that many of the projects discussed in this issue will lead to new drugs and indications.
